# Characterisation of physiological and immunological responses in beef cows to abrupt weaning and subsequent housing

**DOI:** 10.1186/1746-6148-6-37

**Published:** 2010-07-20

**Authors:** Eilish M Lynch, Bernadette Earley, Mark McGee, Sean Doyle

**Affiliations:** 1 Animal and Bioscience Department, Animal & Grassland Research and Innovation Centre, Teagasc, Grange, Dunsany, Co. Meath, Ireland; 2 Department of Biology and National Institute for Cellular Biotechnology, National University of Ireland Maynooth, Co. Kildare, Ireland; 3 Livestock Systems Research Department, Animal & Grassland Research and Innovation Centre, Teagasc, Grange, Dunsany, Co. Meath, Ireland

## Abstract

**Background:**

Weaning involves the permanent separation of the calf from the dam and has been shown to be stressful for both. The objectives of this study were to characterise the effect of i) abrupt weaning and ii) subsequent housing on the extended physiological and immunological responses of beef cows. At weaning (day (d) 0, mean age of calf (s.d.) 212 (24.5) d), cows were abruptly separated from their calves and returned to the grazing area. After 35 d at pasture, cows were housed in a slatted floor shed and offered grass silage *ad libitum *plus a mineral-vitamin supplement daily. Rectal body temperature was recorded and blood samples were obtained on i) d 0 (weaning), 2, 7, 14, 21, 28, 35 and subsequently on ii) d 0 (housing), 2, 7, 14 and 21 for physiological, haematological and immunological measurements.

**Results:**

Post-weaning, concentration of cortisol and dehydroepiandrosterone were unchanged (P > 0.05). Rectal body temperature, neutrophil number and neutrophil: lymphocyte ratio increased (P < 0.01) on d 2 compared with pre-weaning baseline. Lymphocyte and neutrophil number decreased (P < 0.05) on d 2 to 7 and d 7 to 21, respectively, compared with pre-weaning baseline. Interferon-γ production decreased (P < 0.05) on d 2 compared with pre-weaning baseline. An increase (P < 0.05) in acute phase proteins, fibrinogen and haptoglobin was evident on d 2 to 35 compared with pre-weaning baseline. Concentration of glucose increased on d 2 to 28, whereas non-esterified fatty acid decreased on d 2 to 35 compared with pre-weaning baseline. Post-housing, concentrations of cortisol, rectal body temperature, total leukocyte number, and glucose were unchanged (P > 0.05). On d 2 post-housing, neutrophil number and neutrophil: lymphocyte ratio increased (P < 0.05), whereas lymphocyte number and concentrations of dehydroepiandrosterone, fibrinogen and non-esterified fatty acid decreased (P < 0.05) compared with pre-housing baseline. Concentration of haptoglobin increased (P < 0.05) on d 14 to 21 post-housing.

**Conclusions:**

A transitory increase in neutrophil number and decrease in lymphocyte number, increased neutrophil:lymphocyte ratio coupled with decreased interferon-γ production, and increased concentration of acute phase proteins indicate a stress response in cows post-weaning, whereas post-housing, changes were less marked.

## Background

Within seasonal grassland-based, spring-calving suckler beef production systems calves are generally allowed continuous and unlimited nursing of the dam for approximately 6 to 8 months until weaning at the end of the grazing season. Husbandry management practices, including weaning and housing, form integral components of these beef production systems, and often expose beef cattle to novel environmental, physical and psychological stressors. Research measuring stress-related variables in cattle has focused on parturition [1], routine handling [2,3], mixing with unfamiliar cattle [4,5], transportation [6,7], restrictive space allowance during indoor housing [8], and weaning of the calf [9]. However, limited research has examined weaning stress in the cow. The effect of weaning on concentration of cortisol in the cow is equivocal with no change [10] and an increase [11] reported. Furthermore, these studies were limited up to 48 h and 6 d post-weaning, respectively. Extended effects of weaning on physiological and immunological responses were found in beef calves [12] but have not been examined in beef cows.

At the end of the grazing season in autumn, weaned cows are typically housed indoors over the winter period [13,14]. In beef cattle, housing research has focused on the effect of varying space allowance [8,15], and floor type [16,17] on production and behavioural responses. There is limited data available on the effects of moving animals from an outdoor grazing environment to indoor accommodation in slatted floor sheds. Previously grazed cows that were tethered during indoor housing had increased concentration of cortisol for up to 7 days post-housing [18]. Research on the extended effect of indoor housing on other stress related variables is warranted.

Therefore, the objectives of the study were to characterise the extended physiological (rectal body temperature, cortisol, dehydroepiandrosterone (DHEA), glucose, non-esterified fatty acid (NEFA), β-hydroxybutyrate (βHB), total leukocyte number and differentials, red blood cell number (RBC), haemoglobin concentration (HGB), haematocrit percentage (HCT)), and immunological (interferon (IFN)-γ production and acute phase proteins) responses in beef cows to i.) abrupt weaning and ii.) subsequent housing.

## Results

### Rectal body temperature

Rectal body temperature increased (P < 0.01) on d 2 (mean (s.e.) 38.7 (0.04)°C) compared with pre-weaning baseline (d 0 at weaning: mean (s.e.) 38.4 (0.05)°C). Rectal body temperature did not differ (P > 0.05) on d 7 to d 35 compared with d 0. Post-housing, rectal body temperature did not differ (P > 0.05) from pre-housing baseline (d 0 at housing) (data not shown).

### Cortisol and DHEA

Concentration of cortisol was lower than 7.5 ng/mL in cows at all sampling points during the study. On d 2 post-weaning, concentration of cortisol (mean (s.e) 4.4 (0.34) ng/mL) decreased (P < 0.05) by 24% compared with pre-weaning baseline (mean 5.8 (s.e) 0.39 ng/mL). Concentrations of cortisol did not differ (P > 0.05) from pre-weaning and pre-housing baselines for the remainder of the study (Data not shown). Post-weaning, concentration of DHEA did not differ (P > 0.05) from pre-weaning baseline, whereas following housing, concentration of DHEA decreased (P < 0.05) by 51% on d 2 (mean (s.e.) 1.9 (0.55) ng/mL) to 67% on d 21 (mean (s.e.) 1.3 (0.31) ng/mL) compared with pre-housing baseline (mean (s.e.) 3.9 (0.65) ng/mL). The cortisol: DHEA ratio did not differ (P > 0.05) from pre-weaning and pre-housing baselines at any sampling time-points (Data not shown).

### Haematology

**Figure 1:**
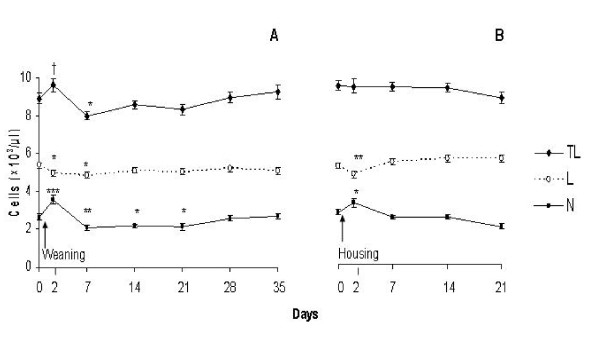
**Effect of abrupt weaning and subsequent housing on total leukocyte, neutrophil, and lymphocyte number in beef cows**. TL = Total leukocyte number, L = lymphocyte number and N = neutrophil number. Significant differences between d 0 (pre-weaning) and d 35 baselines (pre-housing) are denoted by asterisks; * = P < 0.05, ** = P < 0.01 and *** = P < 0.001. There was a tendency for a significant increase (P = 0.07) in total leukocyte number in beef cows at d 2 compared with d 0 and is denoted by †.

**Figure 2:**
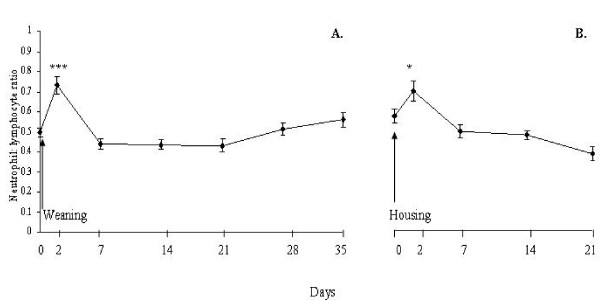
**Effect of abrupt weaning and subsequent housing on neutrophil: lymphocyte ratio in beef cows**. Significant differences between d 0 (pre-weaning) and d 35 baselines (pre-housing) are denoted by asterisks; * = P < 0.05 and *** P < 0.001

Post-weaning, there was a 30% increase (P < 0.001) in neutrophil number with a concurrent 10% decrease (P < 0.05) in lymphocyte number, which resulted in an increase (P = 0.07) in total circulating leukocyte number on d 2 compared with pre-weaning baseline (Figure 1). Subsequently on d 7, total leukocyte number decreased (P < 0.05) and returned to pre-weaning baseline by d 14. This decrease in total leukocyte number reflected the 15 - 22% decrease (P < 0.05) in neutrophil number on d 7, 14 and 21 with a concurrent 11% decrease (P < 0.01) in lymphocyte number on d 7 compared with pre-weaning baseline. The N:L ratio increased (P < 0.001) by 49% on d 2 and returned to pre-weaning baseline on d 7 (Figure 2). Post-housing, neutrophil number increased (P < 0.05) by 18% and lymphocyte number decreased (P < 0.01) by 9% on d 2 and both returned to pre-housing baseline by d 7. Total leukocyte number did not differ (P > 0.05) post-housing compared with pre-housing baseline. The N:L ratio increased (P < 0.05) by 21% on d 2 and returned to pre-housing baseline by d 7 (Figure 2).

Red blood cell number and HCT percentage decreased (P < 0.05) by 6% on d 7 to 21 and by 3% on d 14 and d 21, respectively, compared with pre-weaning baseline. There was no change in HGB concentration post-weaning (Table 1). Post-housing, RBC number was unchanged (P > 0.05), whereas HGB and HCT percentage were increased (P < 0.05) by 2 - 3% on d 7 to 21 compared with pre-housing baseline.

**Table 1:** Least squares means (s.e.) for RBC, HGB, and HCT in beef cows following abrupt weaning and subsequent housing

	**Pre-weaning**	**Days post-weaning**						**Pre-housing**	**Days post-housing**			
		
**Variable**	**0**	**2**	**7**	**14**	**21**	**28**	**35**	**0**	**2**	**7**	**14**	**21**
	
RBC	7.4	7.5	7.2^a^	6.8^a ^	6.9^a^	7.1	7.6	7.6	7.7	7.7	7.5	7.6
(× 10^6^/μl)	(0.10)	(0.09)	(0.11)	(0.07)	(0.08)^a^	(0.08)	(0.11)	(0.11)	(0.09)	(0.10)	(0.09)	(0.09)
HGB	11.9	12.1	12.0	11.6	11.9	12.1	12.0	13.3	13.4	13.6^d^	13.7^d^	13.7^d^
(g/dl)	(0.12)	(0.12)	(0.14)	(0.12)	(0.11)	(0.10)	(0.16)	(0.16)	(0.12)	(0.14)	(0.13)	(0.14)
HCT	31.6	31.9	31.0	30.2^a^	30.6^a^	31.9	34.1^a^	34.1	34.4	35.1^d^	32.1^d^	32.8^d^
(%)	(0.31)	(0.31)	(0.41)	(0.32)	(0.28)	(0.26)	(0.42)	(0.42)	(0.32)	(0.37)	(0.32)	(0.35)

^a,b,c^Within a row, means differ from pre-weaning baseline by P < 0.05, P < 0.01, and P < 0.001, respectively. d,e,fWithin a row, means differ from pre-housing baseline by P < 0.05, P < 0.01, and P < 0.001, respectively. RBC: red blood cell number, HGB: haemoglobin concentration, HCT: haematocrit percentage.

### *In vitro *lymphocyte production of IFN-γ

Following weaning, phytohaemagglutinin (PHA)-induced *in vitro *lymphocyte IFN-γ production decreased (P < 0.05) by 51% on d 2 (mean (s.e.) 0.17 (0.03) OD at 450nm) and by 41% d 35 (mean (s.e.) 0.20 (0.04)) compared with pre-weaning baseline (mean (s.e.) 0.34 (0.05)). The concanavalin A (Con A)-induced production of IFN-γ decreased (P < 0.05) by 49% on d 35 (mean (s.e) 0.30 (0.03)) compared with pre-weaning baseline (mean (s.e.) 0.59 (0.08)). Post-housing, IFN-γ production was unchanged (P > 0.05) for both mitogens (Con A: mean (s.e.) 0.23 (0.04), and PHA: mean (s.e.) 0.24 (0.04)) on d 2 compared with pre-housing baseline (Con A: mean (s.e.) 0.29 (0.03), and PHA: mean (s.e.) 0.20 (0.4)). On d 21, Con A-induced IFN-γ production increased by 110% (P < 0.05; mean (s.e.) 0.58 (0.06)) compared with pre-housing baseline (mean (s.e.) 0.30 (0.03)), whereas PHA-induced IFN-γ production was unchanged (P > 0.05) from pre-housing baseline.

### Acute phase proteins

Concentrations of fibrinogen and haptoglobin increased (P < 0.05) by 16% and 21%, respectively, on d 2 to 35 compared with pre-weaning baseline (Table 2). Post-housing, concentration of fibrinogen decreased (P < 0.05) by 12% on d 2 and subsequently increased (P < 0.001) by 25% on d 14 and decreased (P < 0.001) by 22% on d 21 compared with pre-housing baseline. Concentration of haptoglobin was unchanged on d 2 and 7 (P > 0.05) but decreased (P < 0.05) by 25% on d 14 and 21 compared with pre-housing baseline (Table 2).

**Table 2:** Least squares means (s.e.) for concentration of fibrinogen and haptoglobin in beef cows following abrupt weaning and subsequent housing

	**Pre-weaning**	**Days post-weaning**						**Pre-housing**	**Days post-housing**			
		
**Variable**	**0**	**2**	**7**	**14**	**21**	**28**	**35**	**0**	**2**	**7**	**14**	**21**
	
Fibrinogen	408	458^a^	489^a^	489^a^	493^a^	462^a^	467^a^	467	411^d^	488	587^f^	364^f^
(mg/dl)	(15.2)	(16.9)	(18.6)	(20.7)	(15.7)	(12.8)	(20.1)	(20.1)	(20.1)	(22.2)	(24.9)	(27.2)
Haptoglobi	0.33	0.43^b^	0.53^b^	0.72^b^	0.46^b^	0.57^b^	0.67^b^	0.68	0.74	0.68	0.49^d^	0.54^d^
n (mg/dl)	(0.013)	(0.031)	(0.02)	(0.07)	(0.01)	(0.025)	(0.03)	(0.038)	(0.03)	(0.03)	(0.01)	(0.06)

^a,b,c^Within a row, means differ from pre-weaning baseline by P < 0.05, P < 0.01, and P < 0.001, respectively. d,e,fWithin a row, means differ from pre-housing baseline by P < 0.05, P < 0.01, and P < 0.001, respectively.

### Glucose, βHB and, NEFA

Post-weaning, concentration of glucose increased (P < 0.001) by 16% on d 2 to d 35 compared with pre-weaning baseline. Concentration of βHB increased (P < 0.001) by 34% on d 2, followed by a 21% decrease (P < 0.001) on d 7, and returned to pre-weaning baseline on d 14 (Table 3). Concentration of NEFA decreased (P < 0.05) by 57% on d 2 to 28 compared with pre-weaning baseline. Post-housing, concentration of glucose did not differ (P > 0.05) from pre-housing baseline, whereas concentration of βHB decreased (P < 0.001) by 21% on d 7 and 14 and concentration of NEFA decreased (P < 0.05) by 63% on d 7 to 21 compared with pre-housing baseline (Table 3).

**Table 3:** Least squares means (s.e.) for concentration of glucose, NEFA, and βHB in beef cows following abrupt weaning and subsequent housing

	**Pre-weaning**	**Days post-weaning**						**Pre-housing**	**Days post-housing**			
		
**Variable**	**0**	**2**	**7**	**14**	**21**	**28**	**35**	**0**	**2**	**7**	**14**	**21**
	
Glucose	3.2	3.7^c ^	3.8^c^	3.6^c^	3.8^c^	3.7^c^	3.7^c^	3.7	3.7	3.6	3.8	3.8
(mmol/l)	(0.03)	(0.04)	(0.04)	(0.03)	(0.04)	(0.04)	(0.06)	(0.06)	(0.06)	(0.05)	(0.04)	(0.07)
NEFA	0.37	0.20^a^	0.24^a^	0.12^a^	0.10^a^	0.12^a^	0.41	0.41	0.38	0.26^d^	0.08^d^	0.10^d^
(mmol/l)	(0.08)	(0.09)	(0.08)	(0.008	(0.006	(0.007	(0.08)	(0.008)	(0.008	(0.007)	(0.006)	(0.008
βHB	0.29	0.39^c^	0.23^c^	0.30	0.28	0.29	0.28	0.28	0.27	0.20^f ^	0.24^e^	0.31
(mmol/l)	(0.009)	(0.012	(0.006	(0.010	(0.009	(0.006	(0.008	(0.018)	(0.010	(0.005)	(0.008)	(0.009

^a,b,c^Within a row, means differ from pre-weaning baseline by P < 0.05, P < 0.01, and P < 0.001, respectively. d,e,fWithin a row, means differ from pre-housing baseline by P < 0.05, P < 0.01, and P < 0.001, respectively. NEFA: non esterified fatty acid, βHB: β-hydroxybutyrate.

## Discussion

To our knowledge, no other study has characterised the extended physiological, haematological and immunological responses in beef cows to abrupt weaning or subsequent housing. The effect of post-weaning management practice [previously grazed calves, were abruptly weaned (mean age of calf (s.d.) 212 (24.5) d), and either immediately housed in a slatted floor shed or returned to the grazing area for 35 d and then housed] on physiological and immunological responses in the progeny of the cows used in the present study was also measured up to d 35 post-weaning and d 21 post-housing [12]. Behavioural research to date has shown that breaking the mother-offspring bond alone represents a stressful experience for both mother and young [19]. Following separation from their calves, beef cows generally exhibit similar, less intense, reinstatement behaviours (increased locomotor activity and vocalisations) to their young, in an effort to reunite [20]. Consequently, no behavioural measurements were examined as part of this study.

The milk yield of the cow breed type (beef × dairy) used in the present study remains relatively high at weaning [21] and milk would have constituted a significant proportion of the calves' total diet. There is no information in the literature on the extent to which drying off induces a systemic inflammatory response in beef cows, however, there is considerable literature on systemic and local inflammatory responses during mastitis in dairy cows [22-26]. During the drying off period, the mammary gland continues to synthesise and secrete milk which accumulates in the gland. An increase in intra-alveolar pressure as a result of milk accumulation is thought to trigger involution. Involution-associated ultrastructural changes in bovine mammary cells are reported to commence within 48 hours after cessation of milk removal [27,28] and by day 28 the collapsed alveolar structures remaining are considerably smaller than during lactation. In cows, the alveolar structure is maintained throughout involution and there is no evidence for extensive tissue degeneration that is found in other species, such as rodents [29,30]. The impact of mammary cell apoptosis in the bovine is not fully characterised. In the present study, all animals were monitored daily and no signs of ill-health were recorded which suggests that there was no underlying systemic infection. The small increase in rectal body temperature recorded post-weaning was not of clinical significance [31].

In accord with the findings of [10], where cows were separated from their calves when calves were 4 to 6 months of age, weaning was not associated with increased plasma cortisol concentration in cows. In contrast, [11] using more frequent blood sampling collection time points relative to cow-calf separation than those used in our study, these authors reported increased concentration of cortisol in cows that were separated from their calves 35 days post-partum. The large difference in age at weaning may account for the disparity in cortisol response observed between these studies. The absence of an effect of housing on cortisol concentration contrasts with the findings of [18] who reported an increase in concentration in previously grazed cows that were housed. However, in the latter study, cows were tethered, whereas in the present study cows were loose-housed in pens. In the present study, concentration of DHEA was not affected by weaning. This hormone is episodically co-secreted with cortisol from the adrenal gland and is also a potential precursor for androgen and oestrogen synthesis in cattle [32]. Post-housing, concentration of DHEA decreased without a concomitant increase in cortisol. Increased cortisol: DHEA ratio was reported in lame dairy cows [33] and following transportation of young bulls [34] and was suggested as a potential biomarker of stress. The unaltered cortisol: DHEA ratio found in the present study suggests that the practice of weaning and housing were insufficient to elicit a physiological stress response, whereby a shift occurred in the steroidogenic pathway towards cortisol at the expense of DHEA. Further research investigating the use of cortisol: DHEA ratio as a biomarker of stress is warranted.

Peripheral blood cells are sensitive indicators of patho-physiological responses and may be used to define subclinical disease states in cattle [35]. Alterations in circulating leukocyte subsets have been documented in beef cattle under various management practices such as restricted space allowance during housing of steers [15], transportation of bulls [7], and weaning of calves [12,36]. In agreement with the latter studies using calves, increased neutrophil number and decreased lymphocyte number (increased N:L ratio), was evident in cows post-weaning. The magnitude of the increase in neutrophil number was higher (33% versus 18%) on d 2 post-weaning compared with d 2 post-housing in cows. The changes observed in red blood cell number, haemoglobin concentration and haematocrit percentage in cows were negligible suggesting that there were no negative consequences of weaning or housing on these variables.

The decrease in IFN-γ production on d 2 compared with pre-weaning baseline was consistent with the findings of [12] who weaned calves and returned them to pasture and with [9] and [12] using weaned calves that were immediately housed. In the present study, the relative decrease in PHA-induced IFN-γ production on d 2 post-weaning may be a consequence of decreased lymphocyte number. An absence of a decrease in PHA-induced and Con A-induced IFN-γ production in cows on d 2 post-housing suggests that housing was insufficient to elicit a stress response, and these findings are consistent with [12] who reported unchanged IFN-γ production post-housing in calves that were previously grazed. While lymphocyte number was decreased on d 2 post-housing, in the present study, there was no associated reduction in PHA-induced or Con A-induced IFN-γ production, suggesting that the function of the cells to produce IFN-γ was not impaired.

Overall, these results suggest that cows were more sensitive to the effects of weaning than to the effects of housing due to the less marked neutrophil response and unchanged IFN-γ production observed post-housing. Cows may also be less sensitive to weaning- and housing-associated stress, as indicated by the relatively lower change in neutrophil number and IFN-γ production, compared with their calves [12].

Post-weaning, cows had increased concentrations of plasma fibrinogen and haptoglobin, which is in agreement with the results obtained for calves following abrupt weaning [12]. Following housing, no clear trend was evident for the acute phase protein response. The response post-housing may have been influenced by the change in dietary crude protein concentration.

Blood metabolite concentrations indicate the extent of metabolism of energy, protein and other nutrients and thus, can provide information on the nutritional status of cattle. Typically, stressful events result in reduced feed intake and depletion of energy stores in cattle [31]. In the present study, weaning and subsequent housing caused transient changes in blood metabolites. Increased concentration of glucose is often attributed to glycogenolysis, which is associated with increased catecholamines and glucocorticoid secretion at the onset of a stressor [37]. However, due to the unaltered cortisol response throughout the study, it is unlikely that the increased concentration of glucose is solely a consequence of weaning as a stressor, but rather an indication of a more positive nutritional status in the cow due to the cessation of nursing. Generally, higher concentrations of NEFA and βHB and lower concentrations of glucose are associated with negative energy balance in beef cows [38]. Thus, the opposing trend of concentrations of plasma glucose and NEFA suggests that removal of the calf from the cow resulted in a more positive nutritional state in the cow.

Under the conditions of the present study, animal was the experimental unit and was specified as a repeated measures effect in the statistical analysis and most of the physiological and immunological variables which changed as a consequence of i) weaning and ii) housing had recovered to baseline values on completion of the study. In the present study, while changes were found in physiological and immunological variables in response to weaning and subsequent housing, the values were within the normal physiological ranges for cattle [35,39-41].

## Conclusions

In conclusion, abrupt weaning results in neutrophilia and lymphopaenia coupled with reduced *in vitro *lymphocyte production of IFN-γ and increased acute phase protein response suggesting that weaning elicited a transitory stress response in first parity beef cows. Post-housing, transitory neutrophilia and lymphopaenia were evident, however unchanged total leukocyte number and IFN-γ production and the lack of a clear trend in the acute phase protein response indicates that housing did not elicit a stress response as great as weaning in the beef cow.

## Methods

### Animal management

All animal procedures performed in this study were conducted under experimental licence from the Irish Department of Health and Children in accordance with the Cruelty to Animals Act 1876 and the European Communities (Amendment of Cruelty to Animals Act 1876) Regulation 2002 and 2005.

Thirty-six first parity, spring-calving (mean date of calving (s.d) March, 19 (25.5) d; mean age at calving (s.d.) 756 (24.3) d), single-suckled Limousin × Holstein-Friesian (n = 18) and Simmental × Holstein-Friesian (n = 18) cows were used. Cows and their progeny were rotationally grazed on a predominantly perennial ryegrass (*Lolium perenne*)-based sward from early April until weaning on 17 October. The pasture had a mean (s.d.) dry matter digestibility (DMD) of 750 (22.1) g/kg and mean (s.d.) crude protein (CP) concentration of 229 (8.7) g/kg DM. On the day of weaning (day (d) 0), cows and calves were moved to a handling yard where the cows were abruptly separated from their calves (mean age (s.d.) 212 (24.5) d; mean weight (s.d.) 279 (38.0) kg) and were returned to the grazing area for a 35 day (d) period. The grazing area was located at a sufficient distance away from the handling yard thus, vocalisations between the cows and calves could not be heard. On d 35, the cows were housed indoors in a slatted floor shed in 6 pens (n = 6 cows per pen). Each pen was equipped with automatic water drinkers and cows were offered grass silage *ad libitum *(mean (s.d) DMD 714 (17.7) g/kg; CP 144 (11.3) g/kg DM) plus 60 g of a mineral vitamin supplement daily. Forage analysis methodology was carried out according to [42].

### Blood collection

**Figure 3:**
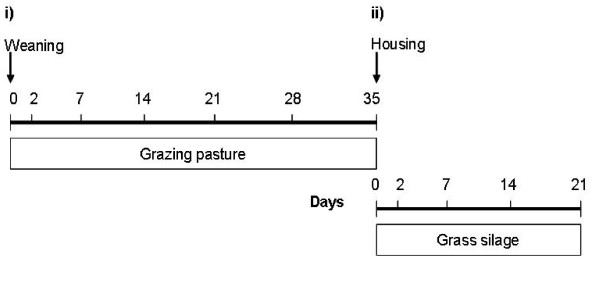
**Management and blood sampling schedule**. Management and blood sampling schedule for beef cows that were abruptly separated from their calves and i) returned to pasture on d 0, and ii) subsequently housed in a slatted floor shed on d 35. Sampling occurred at 7 d intervals except for immediately post-weaning and post-housing where a 2 d interval was allowed for sampling.

Blood samples were collected from the cows by direct jugular venipuncture to evaluate i) the effects of weaning (d 0 (pre-weaning baseline), 2, 7, 14, 21, 28, and 35) and ii) the effects of housing (d 0 (pre-housing baseline), 2, 7, 14 and 21) (Figure 3). Blood (10 mL) samples collected into blood tubes (Vacuette, Cruinn Diagnostics, Ireland) containing lithium heparin were used to determine plasma concentrations of cortisol, DHEA, haptoglobin, glucose, NEFA, βHB, and for the determination of *in vitro *lymphocyte production of IFN-γ from a whole blood culture. Blood samples were collected by direct venipuncture on d 0 (weaning) and 2, and on d 0 (housing) and 2 and 21 for the determination of *in vitro *lymphocyte production of IFN-γ. Blood (4 mL) was collected into vacutainer tubes containing sodium citrate for the subsequent assay of plasma fibrinogen concentration. An additional 6 mL of blood was collected into vacutainer tubes containing K_3_EDTA for haematological determination. All samples were transported to the laboratory at ambient temperature and processed within 2 h of collection.

### Rectal body temperature

Rectal body temperature was recorded prior to blood sampling, as previously described, using a digital centigrade (°C) thermometer (Jørgen Kruuse A/S model VT-801 BWC, Marslev, Denmark; catalogue No. 0701).

### Cortisol and DHEA, acute phase proteins, Glucose, βHB and NEFA

Plasma was harvested from lithium heparin and sodium citrate anti-coagulated blood following centrifugation at 1600 × *g *at 4°C for 15 min and stored at -80°C until assayed for cortisol, DHEA, haptoglobin and fibrinogen, glucose, βHB and NEFA. Plasma cortisol and DHEA were assayed using the Correlate-EIA kits from Assay Designs (Ann Arbor, MI, USA). The mean intra-assay CV (n = 5) for cortisol and DHEA was 8.7% and 5.3%, respectively. The corresponding mean inter-assay CV (n = 8) was 6.9% and 4.5%. Assay sensitivity for cortisol was 56.7 pg/mL and for DHEA was 2.9 pg/mL. The ratio of cortisol: DHEA was calculated. Concentration of plasma haptoglobin was measured using an assay kit (Tridelta Development Ltd., Wicklow, Ireland) on an automatic analyser (spACE, Alfa Wassermann, Inc., West Caldwell, NJ, USA) according to the procedure of [43]. Concentration of plasma fibrinogen was measured according to the method of [44] on an automated analyser (spACE, Alfa Wassermann, Inc., West Caldwell, NJ, USA). Concentrations of plasma glucose, NEFA, and βHB were determined using an automatic analyser (Olympus AU400, Japan) using assay kits supplied by Olympus.

### Haematology

Total circulating leukocyte number, neutrophil and lymphocyte number, RBC number, concentration of HGB, HCT percentage were determined from K_3_EDTA anti-coagulated whole blood using an automatic haematology analyser (AV ADIVA 2120, Bayer Healthcare, Siemens, UK) equipped with software for bovine blood. Neutrophil: lymphocyte (N:L) ratio was calculated.

### *In vitro *production of interferon-γ

A whole blood culture procedure [45] was used to determine the *in vitro *lymphocyte production of interferon-γ (IFN- γ) in lithium heparinised whole blood. Duplicate 1.48mL aliquots of blood were cultured in sterile 24 well flat culture plates (Sarstedt Ltd., Drinagh, Wexford, Ireland) with 20 μL of phosphate buffer saline (PBS) (GibcoBRL, Life Technologies Ltd., Paisley, Scotland, UK) containing 1.0 mg/mL of concanavalin A (Con A) (Sigma-Aldrich, Inc., UK), 1.0 mg/mL phytohaemagglutinin (PHA) (Sigma-Aldrich, Inc., UK) or no additive, for 24 h at 37°C and in an atmosphere of 5% CO_2_. The culture plates were then centrifuged at 1600 × g at 4°C for 20 min, supernatant harvested and frozen at -20°C until assayed for INF-γ using an ELISA procedure specific for bovine plasma (BOVIGAM, Biocor Animal Health, NE, USA), as previously described [46]. The *in vitro *Con A or PHA stimulated production of IFN-γ was calculated by subtracting the absorbance at 450 nm of wells that received PBS alone from wells that received Con A or PHA, respectively.

## Statistical analysis

Data was analysed as 2 separate datasets relative to management practice: i.) weaning (d 0 to d 35), and ii.) subsequent housing (d 0 to d 21). Each dataset was analysed as repeated measures using the PROC MIXED procedure of SAS (Version 9.1, SAS Institute, Cary, NC) with an unstructured covariance matrix within animal. Breed (Limousin × Holstein-Friesian and Simmental × Holstein-Friesian) and sampling time were listed in the model statement. Breed was not significant and was excluded from the final model. Least squares means were estimated and differences between least squares means were tested using the PDIFF option in SAS. A probability of P < 0.05 was chosen as the level of significance.

## Authors' contributions

BE and MMcG designed the study. EL and BE performed the experiments. BE and EL analysed the data and EL prepared the manuscript. EL, BE, MMcG and SD contributed to, read and approved the final manuscript.

## References

[B1] Burton JL Madsen SA Chang LC Weber PSD Buckham KR van Dorp R Hickey MC Earley B Gene expression signatures in neutrophils exposed to glucocorticoids: A new paradigm to help explain "neutrophil dysfunction" in parturient dairy cows Vet Immunol Immunopathol 2005 105 197 219 10.1016/j.vetimm.2005.02.012 15808301

[B2] Grignard L Boivin X Boissy A Le Neindre P Do beef cattle react consistently to different handling situations Appl Anim Behav Sci 2001 71 263 276 10.1016/S0168-1591(00)00187-8 11248377

[B3] Breuer K Hemsworth PH Coleman GJ The effect of positive and negative handling on the behavioural and physiological responses of nonlactating heifers Appl Anim Behav Sci 2003 84 3 22 10.1016/S0168-1591(03)00146-1

[B4] Gupta S Earley B Ting STL Crowe MA Effect of repeated regrouping and relocation on the physiological, immunological, and haematological variables and performance of steers J Anim Sci 2005 83 1948 1958 16024716 10.2527/2005.8381948x

[B5] Raussi S Boissy A Delval E Pradel P Kaihilahti J Veissier I Does repeated regrouping alter the social behaviour of heifers? Appl Anim Behav Sci 2005 93 1 12 10.1016/j.applanim.2004.12.001

[B6] Marahrens M Von Richthofen I Schmeiduch S Hartung J Special problems with long-distance road transports of cattle Dtsch Tierarztl 2003 110 120 125 12731113

[B7] Buckham Sporer KR Burton JL Earley B Crowe MA Transportation stress in young bulls alters expression of neutrophil genes important for the regulation of apoptosis, tissue remodelling, margination, and anti-bacterial function Vet Immunol Immunopathol 2007 118 19 29 10.1016/j.vetimm.2007.04.002 17512060

[B8] Fisher AD Crowe MA O'Kiely P Enwright WJ Growth, behaviour, adrenal, and immune responses of finishing beef heifers on slatted floors at 1.5, 2.0, 2.5 or 3m^2 ^space allowance Livest Prod Sci 1997 51 245 254 10.1016/S0301-6226(97)00052-3

[B9] Hickey MC Drennan M Earley B The effect of abrupt weaning of suckler calves on the plasma concentrations of cortisol, catecholamines, leukocytes, acute phase proteins and *in vitro *interferon-gamma production J Anim Sci 2003 81 2847 2855 14601889 10.2527/2003.81112847x

[B10] Lefcourt AM Elsasser TH Adrenal responses of Angus × Hereford cattle to stress of weaning J Anim Sci 1995 73 2669 2676 8582857 10.2527/1995.7392669x

[B11] Whisnant CS Kiser TE Thompson FN Effect of calf removal on serum luteninizing hormone and cortisol concentration in postpartum beef cows Theriogenology 1985 24 119 129 10.1016/0093-691X(85)90217-1 16726064

[B12] Lynch EM Earley B McGee M Doyle S Effect of weaning strategy on immunological, hematological and physiological responses of beef calves J Anim Sci E Suppl 2008 89 156

[B13] Drennan MJ McGee M Performance of spring-calving beef suckler cows and their progeny on four contrasting grassland management systems Livest Sci 2008 117 238 248 10.1016/j.livsci.2007.12.018 PMC7102801 32288868

[B14] Drennan MJ McGee M Performance of spring-calving beef suckler cows and their progeny to slaughter on intensive and extensive grassland management systems Livest Sci 2009 120 1 12 10.1016/j.livsci.2008.04.013 PMC7102801 32288868

[B15] Gupta S Earley B Crowe MA Pituitary, adrenal, immune and performance of mature Holstein × Friesian bulls housed on slatted floors at various space allowances Vet J 2007 173 594 604 10.1016/j.tvjl.2006.02.011 16647872

[B16] Lowe DE Steen RWJ Beattie VE Moss BW The effect of floor type systems on the performance, cleanliness, carcass composition and meat quality of housed finishing beef cattle Livest Prod Sci 2001 69 33 42 10.1016/S0301-6226(00)00246-3

[B17] Platz S Ahrens F Bahrs E Nuske S Erhard MH Association between floor type and behaviour, skin lesions, and claw dimensions in group-housed fattening bulls Preventive Veterinary Medicine 2007 80 209 211 10.1016/j.prevetmed.2007.02.007 17383035

[B18] Higashiyama Y Nashiki M Narita H Kawasaki M A brief report on effects of transfer from outdoor grazing to indoor tethering and back on urinary cortisol and behaviour in dairy cattle Appl Anim Sci 2007 102 119 123 10.1016/j.applanim.2006.03.007

[B19] Newberry RC Swanson JC Implications of breaking mother-young social bonds Appl Anim Behav Sci 2008 110 3 23 10.1016/j.applanim.2007.03.021

[B20] Price EO Harris JE Borgwardt RE Sween ML Connor JM Fenceline contact of beef calves with their dams at weaning reduces the negative effects of separation on behaviour and growth rate J Anim Sci 2003 81 116 121 12597380 10.2527/2003.811116x

[B21] McGee M Drennan MJ Effect of suckler cow genotype on milk yield and pre-weaning calf performance Ir J Agric Food Res 2005 44 185 194

[B22] Nickerson SC Immunological aspects of mammary involution J Dairy Sci 1989 72 1665 1678 10.3168/jds.S0022-0302(89)79278-X 2668362

[B23] Sordillo LM Shafer-Weaver K DeRosa D Immunobiology of the mammary gland J Dairy Sci 1997 80 1851 1865 10.3168/jds.S0022-0302(97)76121-6 9276826

[B24] Sordillo LM Factors affecting mammary gland immunity and mastitis susceptibility Livest Prod Sci 2005 98 89 99 10.1016/j.livprodsci.2005.10.017

[B25] Singh K Dobson J Phyn CVC Davis SR Farr VC Molenaar AJ Stelwagen K Milk accumulation decreases expression of genes involved in cell-extracellular matrix communication and is associated with induction of apoptosis in the bovine mammary gland Livest Prod Sci 2005 98 67 78 10.1016/j.livprodsci.2005.10.016

[B26] Rainard P Riollet C Innate immunity of the bovine mammary gland Vet Res 2006 37 369 400 10.1051/vetres:2006007 16611554

[B27] Holst BD Hurley WL Nelson DR Involution of the bovine mammary gland: Histological and ultrastructural changes J Dairy Sci 1987 70 935 944 10.3168/jds.S0022-0302(87)80097-8 3597934

[B28] Hurley WL Mammary gland function during involution J Dairy Sci 1989 72 1637 1646 10.3168/jds.S0022-0302(89)79276-6 2668360

[B29] Walker NI Bennett RE Kerr JF Cell death by apoptosis during involution of the lactating mammary breast in mice and rats Am J Anat 1989 185 19 32 10.1002/aja.1001850104 2782275

[B30] Huang RY Ip MM Differential expression of integrin mRNAs and proteins during normal rat mammary gland development and in carcinogenesis Cell Tissue Res 2001 303 69 80 10.1007/s004410000293 11236006

[B31] Duff GC Galyean ML Board-Invited Review: Recent advances in management of highly stressed, newly received feedlot cattle J Anim Sci 2007 85 823 840 10.2527/jas.2006-501 17085724 PMC7109667

[B32] Marinelli L Trevisi E Da Dalt L Merlo M Bertoni G Gabai G Dehydroepiandrosterone secretion in secretion in dairy cattle is episodic and unaffected by ACTH stimulation J Endocrinol 2007 194 627 635 10.1677/JOE-07-0226 17761902

[B33] Almeida PE Weber PSD Burton JL Zanella AJ Depressed DHEA and increased sickness response behaviours in lame dairy cows with inflammatory foot lesions Dom Anim Endocrinol 2007 34 89 99 10.1016/j.domaniend.2006.11.006 17229542

[B34] Buckham Sporer KR Weber PSD Burton JL Earley B Crowe MA Transportation of young beef bulls alters circulating physiological parameters that may be effective biomarkers of stress J Anim Sci 2008 86 1325 1334 10.2527/jas.2007-0762 18344301

[B35] Jones ML Allison RW Evaluation of the ruminant complete blood cell count Vet Clin North Am Food Anim Pract 2007 23 377 402 10.1016/j.cvfa.2007.07.002 17920454

[B36] Blanco M Casasús I Palacio J Effect of age at weaning on the physiological stress response and temperament of two beef cattle breeds Animal 2009 3 108 117 10.1017/S1751731108002978 22444177

[B37] McDowell GH Hormonal control of glucose homeostasis in ruminants Proc Nutr Soc 1983 42 149 167 10.1079/PNS19830021 6351077

[B38] McGee M Drennan MJ Caffrey PJ Effect of suckler cow genotype on energy requirements and performance in winter and subsequently at pasture Ir J Agr Food Res 2005 44 157 171

[B39] Radostisis OM Gay CC Blood DC Hinchcliff KW Veterinary Medicine: A textbook of the diseases of cattle, sheep, pigs, goats and horses 2000 9 London: WB Saunders

[B40] Earley B Crowe MA Effects of ketoprofen alone or in combination with local anaesthesia during the castration of bull calves on plasma cortisol, immunological, and inflammatory responses J Anim Sci 2002 80 1044 1052 12002311 10.2527/2002.8041044x

[B41] Kramer JW Feldman BF, Zinkl JG, Jain NC Normal haematology of cattle, sheep, and goats Schalm's Veterinary Haematology 2006 5 Oxford: Blackwell Publishing Ltd 1075 1084

[B42] Owens D McGee M Boland T O'Kiely P Rumen fermentation, microbial protein synthesis, and nutrient flow to the omasum in cattle offered corn silage, grass silage, or whole-crop wheat J Anim Sci 2009 87 658 668 10.2527/jas.2007-0178 18952732

[B43] Eckersall PD Duthie S Safi S Moffatt D Horadagoda NU Doyle S Parton R Bennett D Fitzpatrick JL An automated biochemical assay for haptoglobin: prevention of interference from albumin Comp Haematol Int 1999 9 117 124 10.1007/BF02600369

[B44] Becker U Bartl K Wahlefed AW A functional photometric assay for plasma fibrinogen Thromb Res 1984 35 475 484 10.1016/0049-3848(84)90280-9 6484895

[B45] Wood PR Corner LA Plackett P Development of a simple, rapid *in vitro *assay for bovine tuberculosis based on the production of gamma interferon Res Vet Sci 1990 49 46 49 2116655

[B46] Rothel JS Jones SL Corner LA Cox JC Wood PR A sandwich enzyme immunoassay for bovine interferon-gamma and its use for the detection of tuberculosis in cattle Aust Vet J 1990 67 134 137 10.1111/j.1751-0813.1990.tb07730.x 2115767

